# Yoga and Mindfulness as Therapeutic Interventions for Stroke Rehabilitation: A Systematic Review

**DOI:** 10.1155/2013/357108

**Published:** 2013-05-27

**Authors:** Asimina Lazaridou, Phaethon Philbrook, Aria A. Tzika

**Affiliations:** ^1^NMR Surgical Laboratory, Department of Surgery, Division of Burns, Massachusetts General Hospital and Shriners Burn Institute, Harvard Medical School, Boston, MA 02114, USA; ^2^Radiology, Athinoula A. Martinos Center for Biomedical Imaging, Harvard Medical School, Boston, MA 02114, USA

## Abstract

*Aim*. This paper reports a systematic review and critical appraisal of the evidence on the effectiveness of behavioral therapies such as yoga and mindfulness practices for stroke rehabilitation. *Background*. The experience of stroke can have a negative impact on both psychological and physical health and on quality of life. Yoga and relevant practices are promising therapies that have been used with patients with a variety of conditions. In order to draw conclusions on effectiveness for stroke patients, the evidence requires systematic assessment. *Methods*. A comprehensive search of major biomedical and complementary medicine databases was conducted. Relevant research was categorized by study type and appraised according to study design. *Results*. Five randomized controlled clinical trials and four single case studies were found. Additionally, one qualitative research study was identified. Studies reported positive results, including improvements in cognition, mood, and balance and reductions in stress. Modifications to different yoga practices make comparison between studies difficult, and a lack of controlled studies precludes any firm conclusions on efficacy. *Conclusion*. Yoga and mindfulness could be clinically valuable self-administered intervention options for stroke rehabilitation. Further research is needed to evaluate these specific practices and their suitability in stroke rehabilitation.

## 1. Introduction

Stroke is one of the most prevalent diseases worldwide causing devastating impairments and negative consequences for survivors [1]. Moreover, it is a main cause of adult-onset disability among people and the cost for care is among one of the fastest-growing Medicare expenses [2]. Poststroke therapy may improve recovery and reduce long-term disability [3], but more psychological therapies for evaluating the specific effects of rehabilitation are needed. Given that many rehabilitation programs currently offer yoga as an option to patients, and that yoga is included as a therapeutic option in a number of rehabilitation medicine texts [4–6], a systematic review of its importance warrants further investigation. 

Yoga and mindfulness can be regarded as a main form of alternative medicine therapy [7]. Yoga is an ancient tradition coming from the Sanskrit word “yoga” meaning union or one-pointed awareness. In the Yoga sutras, Patanjali defined the word “yoga” in the first sutra as Atha yoga anushasanam, which means “yoga” is a form of discipline [8]. The word “anushasan” can be broken down into two parts: “anu” meaning “the subtle aspects of human personality,” and “shasan” meaning to “rule over” or to “govern” [9]. Therefore, the concept of yogic discipline is knowledge of the subtle dimensions, the aspects of human personality and directing or governing the subtle nature. In the absence of this discipline there will always be a search to find happiness and harmony, a persistent sense of emptiness inside, and a feeling of not fulfilling or deriving the best from life. Yoga practices foster willpower, discipline, and self-control and force the mind and body to work in perfect synergy. Therefore, yoga exercises may have beneficial effects as a stand-alone treatment on stress reduction and overall well-being [10, 11]. In addition, yoga has been seen as a main discipline and practice that has the potential to cultivate mindfulness [12]. However, most literature has focused on mindfulness that is developed through yoga meditation [13], a self-regulation practice that focuses on training attention and awareness in order to exhibit a mental process that reinforces mental health well-being and mental stability. 

Dr. John Kabat Zinn, in late 1970, while teaching mindfulness and hatha yoga in Boston, noticed that his trainees were seeking both hatha yoga practices, including asanas (physical exercises), and mindfulness meditation. Therefore, he and his colleagues developed a clinical service that used relatively intensive training in mindfulness meditation practices based on the Vipassana and Zen traditions, along with hatha yoga, for medical patients suffering from a wide range of chronic disorders and diseases [14]. This program evolved into an 8-week course, now known as mindfulness-based stress reduction (MBSR), which is taught worldwide in different centers internationally [15]. Noticeably, practicing mindfulness meditation does not confute the practices of yoga [16]. One practice acts complementary to the other depending on how it is taught and what the needs of the trainees are. 

Mindfulness can be defined as a cognitive process that employs the creation of new categories, openness to new information, and awareness of more than one perspective [17]. Dr. Hirst suggests that being mindful requires the person to attend, to be consciously aware of the emergent nature of phenomena in consciousness, and to recognize the nature of attachments made to these phenomena as they occur [18]. Mindfulness, according to Dr. Kabat-Zinn et al. [19], is based on Eastern contemplative tradition and involves “bringing one's attention to the present experience on a moment-by-moment basis” [14, 15]. On the other hand, Professor Langer discusses the cognitive model of mindfulness without emphasis on the meditative part [17]. She believes that mindfulness could be easier understood with the opposite concept: a state of being as if on automatic pilot, involving preoccupation, absent mindedness, carelessness, in attention, disassociation from feelings, thoughts, actions, and habitual responses. Meanwhile, there is now considerable evidence of the effectiveness of mindfulness-based interventions at reducing distress [20, 21] and rehabilitation [22, 23]. 

Notably, there are many different kinds of hatha yoga and mainstreams based on the multiple traditions that they follow [4, 11, 24]. Thus, the present review will attempt to address this gap within the literature and synthesize the existing research on the positive effects of yoga and relevant meditative practices on stroke rehabilitation.

## 2. The Present Review

The aim of the present review is to conduct a systematic review of the literature concerning holistic therapies such as yoga and mindfulness as a therapeutic application on stroke rehabilitation. Specifically, only studies evaluating the yoga effects on stroke patients were included.

## 3. Materials and Methods

### 3.1. Overview Methodology

The process used for this literature review was highly structured and comprised a number of distinct phases. First, there was the “searching phase,” which involves the systematic identification of potentially relevant studies. The second phase was the “screening phase,” where a predetermined inclusion and exclusion criteria were applied, allowing us to identify appropriate studies for review. In the third phase, or “data-extraction phase,” studies that met the pre-determined inclusion and exclusion criteria were examined in-depth to assess the quality of the study and extract evidence for synthesis. In the fourth stage, or “synthesis phase,” the authors developed a framework for analyzing the selected materials. Finally, in the “reporting phase,” the authors decide on the most efficient and assessable way to present their findings.

### 3.2. Search Terms

The terms used for stroke were based primarily on those used by the Cochrane Cancer Field [35]. The terms used were stroke and mindfulness, MBSR, yoga, pranayama, dhyana, asanas, yogic, meditation, meditat*, transcendental meditation, or mindfulness. 

### 3.3. Search Databases

Systematic searches included major biomedical, nursing, and specialist complementary therapy databases including MEDLINE, EMBASE, AMED, CISCOM, CINAHL, PsycINFO, PubMed, Web of Science, Science Direct, EBSCO, Scopus British Nursing Index, and the Cochrane Library. A search of specialist resources included Cochrane Complementary Field Registry and other Cochrane Specialist Registries. Search strategies were developed to accommodate the different indexing approaches used by the databases [36]. Efforts were made to identify unpublished and ongoing research using relevant databases such as the National Research Register (UK) and Clinicaltrials.gov (USA) together with contacting experts in the field. Reference lists of relevant articles were reviewed to identify further studies.

### 3.4. Filtering

Potential research papers were noted for retrieval and given a preliminary “study type” classification as systematic reviews, randomized controlled trials (RCT), single case reports, qualitative research, or conferences presentations. Animal and basic laboratory-based studies were not included in the categorization process since these settings require a different design procedure. Two reviewers carried out this process independently, notes were compared, and in cases of disagreement these papers were also retrieved.

### 3.5. Inclusion Criteria

Studies written in English, were conducted between 1990–2013, drawn for published research, used yoga or relative meditative/mindfulness practices as an intervention in stroke rehabilitation, were case control and randomized control trials or cohort studies were included. We included any form of yoga practice, including meditation, pranayama, hatha yoga (which is part of the mindfulness stress reduction program). Posters or oral presentations published in scientific journals were included.

### 3.6. Exclusion Criteria

Studies that were not conducted in English, were conducted before 1990, drawn from unpublished work, used yoga as an intervention for treating other diseases, or were based on a single person's opinion were excluded from the review. 

## 4. Results

No systematic reviews relating specifically to stroke rehabilitation and yoga therapy were identified except for one combining a systematic review and results from a pilot study [26]. In total ten studies were identified meeting the inclusion and exclusion criteria. Out of ten studies, five were randomized control studies [21, 25, 28, 31, 33, 34], four were single case report studies [26, 27, 30, 32], and one was a qualitative study [29]. These studies were conducted in USA (*n* = 5), Canada (*n* = 1), Switzerland (*n* = 1), Saudi Arabia (*n* = 1), and Australia (*n* = 2) between 2003 and 2013. Among them, there were five randomized control trials (RCTs), which employed two arms. Six of these studies used the sham intervention for the control groups. None of the studies used a double-blind design. Interestingly, all studies found and conducted after 2003 discussed the relative novelty of the specific therapy for stroke rehabilitation. After 2010, a growing interest particularly in stroke rehabilitation and development of novel alternative therapies in stroke survivors was noticed. Therefore, yoga and mindfulness techniques seem to be an increasing topic of interest, urging the need for more focused investigations to evaluate their effectiveness. All studies included are presented in Table 1, together with comments on their methodology and clinical relevance. Trials are also further discussed in narrative form in order to illustrate differences between studies and in an attempt to assist in highlighting the issues to be addressed in future directions. It was not possible to combine the results of studies due to the variation in the interventions and outcome measures.

### 4.1. Randomized Controlled Trials

Schmid et al. [25] explored whether an 8-week yoga intervention would impact the rehabilitation of veteran stroke survivors. Even though there were no significant differences between the control and experimental groups in the independent tests, within-group tests showed a significant improvement in balance in the yoga group. Chan et al. [37] applied a 6-week yoga intervention in stroke survivors. Depression and anxiety were measured before and after test. Changes in depression and state and trait anxiety did not significantly differ between interventions. However, comparison of individuals' case results indicated clinically relevant improvements in both groups, with members of the intervention group having a greater improvement. Johansson et al. [31] applied an-8 week mindfulness-based stress reduction program (MBSR) for traumatic brain injury and stroke patients focusing on mental fatigue. After the MBSR, improvements in mental fatigue were found in stroke survivors [31]. John et al. [33] explored whether a 6-week yoga intervention would impact disability, balance, fatigue, and depression. Results indicated that the meditation group improved significantly in all measurements after training.

### 4.2. Single Case Studies

Bastille and Gill-Body [27] examined the effects of a yoga-based therapy intervention on mobility and balance in four stroke patients at least 9 months following stroke. The small number of participants did not allow for statistical analysis on the significance of the findings. However, the authors chose to report the findings in a single subject, before and after study design. The investigators reported improvement on the Berg balance score in two of the participants following the intervention and improvement on the timed movement battery in three of the participants. A measure of improvement was seen as a change of at least two standard deviations from the baseline in each patient. Lynton et al. [26] assigned 3 participants into a Kundalini yoga program for 2 weeks. Dexterity and speech improved significantly after the interventions relative to baseline. However, due to the small sample size, it was impossible to draw definite conclusions, but the positive trends in this study suggest that further research should be conducted. The study designed by McEwen et al. allowed the patients to choose the interventions and goals they wished to set. The three goals selected by one participant were clipping the nails on his left hand by using clippers with his affected right hand, walking while carrying an object in his affected right, hand and learning basic yoga or deep breathing techniques. Improved performance for this participant was maintained at a 1-month followup for all but one goal, yoga [30]. Finally, Hofer et al. [32] applied a treatment for stroke, which was a combination of neuropsychological interventions, psychoeducation, cognitive-behavioral therapy, and mindfulness techniques. For the patients with poststroke fatigue (PSF), the central goal was to learn better coping skills regarding their increased vulnerability to fatigue. The significant changes in the symptoms of PSF as well as the achievement of the individually formulated therapy goals support the notion that mindfulness enhances the adjustment process to PSF.

### 4.3. Published Posters or Oral Presentations

Van Puymbroeck et al. [34] designed a 10-week yoga intervention for improving quality of life in stroke survivors. To measure activity and participation, the International Classification of Functioning, Disability and Health (ICF) measure of participation and activity (IMPACT) subscales were used. To measure quality of life, the stroke survivor's quality of life (SSQOL) scale was distributed to participants. Results showed significantly improved activity, participation, and quality of life in the yoga group compared to the control group.

### 4.4. Qualitative Studies

Garrett et al. [29] conducted a qualitative study exploring participants experiences after a yoga program. After the intervention, participants reported greater sensations, feelings of tranquility, and becoming connected to their body and self. These themes respectively revealed perceived physical improvements in terms of strength, range of movement or walking ability, an improved sense of calmness, and the possibility for reconnecting and accepting a different body. This study implies yoga's positive outcomes and sets the base for future quantitative investigations [29].

## 5. Discussion

The authors of this systematic review suggest that yoga is a useful tool for the rehabilitation process after stroke. Since stroke is a leading disease, the need for effective tools for rehabilitation is vital. All studies in the systematic review focused on mood, fatigue, stress, cognitive ability, and quality of life after stroke. This review shows that yoga and stroke rehabilitation have seldom been addressed. Therefore, this systematic review highlights the lack of definitive evidence of yoga's efficacy in stroke rehabilitation and suggests that this topic warrants future investigation. Methodological limitations of the studies included in this review were small sample sizes, limited descriptions of the randomized process when applicable, lack of reporting sampling methods, reasons for dropouts, and insufficient description of specific yoga or meditative practices.

Focus on cognitive functionality after stroke is suggested for future studies, since stroke patients suffer from cognitive dysfunction. One of the most vital problems when comparing different yoga interventions or mindfulness programs is that they are multimodal interventions with mindfulness as their focus. In a pragmatic trial this might not play a vital role but it does have implications for replication and transferability of the study. Moreover, yoga interventions and MBSR programs applied for stroke survivors so far have had relatively small samples and may be unpowered. Yoga interventions should therefore be designed to meet patients' different characteristics (time after stroke, level of impairment, function, and mobility). Physical changes in the form of improved mobility, motor coordination and cognitive changes in the form of improvement of speech impairments seem to be the main components that stroke survivors could benefit from [26].

Through the methods of body posture, breathing training, and consciousness meditation, overall well-being could be improved with positive benefits to the nervous system [38], endocrine system [39], cardiovascular system [40], respiratory system [41], and immunity [42]. Few studies have demonstrated the effects of mindfulness and yoga on well-being, somatic effects of stress, immune system and physical symptoms and chronic conditions [43, 44]. After meditation practice, results showed that the density of gray matter increased in regions governing distinctly different activities as memory, self-awareness, and compassion. Additionally, grey matter decreased in the amygdala, the part of the brain associated with fear and stress [45]. In a more recent study, relaxation techniques seem to affect the genes involved in controlling how the body handles free radicals, inflammation processes, and cell death [46]. More specifically, relaxation techniques improve mitochondrial energy production and utilization and thus promoting mitochondrial resiliency through the upregulation of ATPase and insulin function [46].

 Interestingly, there was no identified study exploring the neurobiology and plasticity of stroke patients after a yoga or mindfulness-based intervention. There is now considerable evidence of MBSR yoga programs on brain alteration and structural and functional plasticity [45]. Recently, several cross sectional anatomical MRI studies have demonstrated that experienced meditators exhibit a different gray matter morphometry in multiple brain regions suggesting plasticity when compared with nonmeditating individuals [45, 47–52]. Four case control studies recently showed significantly higher levels of selective attention in meditators compared to controls, with some specific differences across trials [37, 53–55]. Other results suggest that mindfulness training may improve attention-related behavioral responses by enhancing functioning of specific subcomponents of attention. Whereas participation in the MBSR course improved the ability to internally orient attention, retreat participation appeared to allow for the development and emergence of receptive attention skills, which improved external alerting-related process. Those findings could have dramatic effects for stroke survivors since cognitive skills (thinking, reasoning, judgment, and memory) are mainly impacted. Emotional liability is another component following stroke where mindfulness could have beneficial effects. Mindfulness training is suggested to decrease cognitive rumination, [56] an important component of self-critical elaboration linked to midline prefrontal cortex (PFC) and reactivity in depression [57–59]. The midline PFC seems to be connected to negative-mood induction [59] and exposure to negative-self beliefs [60]. MBSR programs have been associated with decreased activation of these cortical midline structures [59, 60], and efforts to mindfully attend to experience can reduce cortical midline activity in beginners [59]. Several other studies have observed more extensive reductions in cortical activity during meditation, [45, 61, 62] an effect that appears to increase with greater meditation experience [63]. Thus, it may be deduced that one function of mindfulness training is to reduce negative or self-critical judgment associated with cortical midline activity. Additionally, hatha yoga practices could help in limb rehabilitation in stroke survivors. Mindfulness and yoga practices might improve poststroke hemiparesis, [27] although more focused research is needed to determine their effectiveness.

Finally, these findings are of vital importance since problems of emotional processing, including impaired mood, emotion regulation, and emotion perception, are known to occur following stroke and can detrimentally influence many aspects of social interaction after stroke. We suggest that investigations using magnetic resonance imaging (MRI) and magnetoencephalography (MEG) as biomarkers of cognitive brain functions are needed in stroke survivors. Moreover, targeted interventions such as yoga and mindfulness as forms of cognitive therapy should be used addressing specific patients' needs and contraindications. Yoga and mindfulness interventions are a novel therapeutic approaches to personalized alternative medicine that encourages patients to improve their body and mind health by incorporating both new practices and philosophy in life. 

## 6. Conclusions

Yoga seems to offer a relief from a long list of medical ailments in stroke by alleviating both the mind and the body from stress. Yoga and meditative practices act on both the psychological and physical levels, and improvements have been noticed in patients' mindsets [25]. For example, participants in one study [25] talked about incorporating physical activity in their everyday lives more than they used to after yoga intervention. These changes in the mindsets of people with disease can potentially lead to a change in behavior and ultimately an improvement in health [17]. Therefore, the moderating role of mindset and its ability to enhance health should be identified further, substantiated, and utilized in future directions.

## Figures and Tables

**Figure 1:**
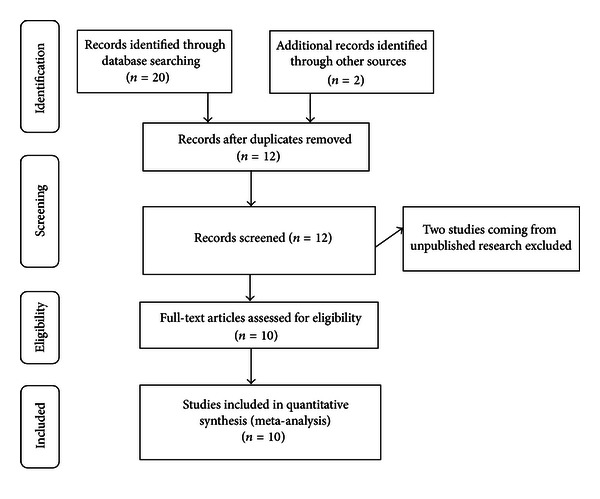
Flow of information through the different phases of a systematic review.

**Table 1:** Yoga as a therapeutic intervention for stroke rehabilitation.

Authors	Groups	Outcome measures	*N*	Sample	Design	Interesting finding
Schmid et al., 2012 [25]	Wait list (control) Yoga	(1) Disability independent (2) Fear of falling (3) Balance (4) Balance self-efficacy (5) QOL	Control *N* = 10 Yoga *N* = 37	Stroke patients Veterans	RCT 8-week intervention	In within-group comparisons, yoga group data demonstrated significant improvement in balance

Lynton et al., 2007 [26]	Yoga	(1) O'Connor tweezer dexterity (2) Boston aphasia exam	*N* = 3	Stroke patients 6 months after stroke Beth Israel Medical Center, NY, USA	Single case study 2 weeks Kundalini yoga practice	All 3 participants showed improvement on dexterity

Bastille and Gill-Body, 2004 [27]	Yoga	(1) Berg balance scale (2) Timed movement battery (3) Stroke impact scale	*N* = 4	Stroke patients <9 months after stroke Keene, NH, USA	Single case study 4–7-week baseline period followed by 8-week intervention yoga practice	3 subjects had improved TMB scores, and 2 subjects had improved BBS scores

Chan and Woollacott, 2007 [28]	Exercise group (control) Yoga exercise group (intervention)	(1) Geriatric depression scale (2) State trait anxiety inventory	Control *N* = 6 Yoga *N* = 8	Poststroke population Royal Adelaide, Hospital South Australia	Single-blinded RCT 6-week standardized program that included home practice	Participants in both groups exhibited a mixture of decreases, increases, and no changes in GDS15, STAI-Yl, and STAI-Y2 over the course of the trial

Garrett et al., 2011 [29]	Wait list (control) Yoga (intervention)	Biopsychosocial model (1) Physiological experiences (2) Psychological experiences	Control (*N* = 12) Yoga (*N* = 10)	Individuals with chronic poststroke hemiparesis 9 months after stroke South Australia	Qualitative RCT 10-week yoga program involving movement, breathing, and meditation practices	Participants reported greater sensation, feeling calmer, and becoming connected

McEwen et al., 2009 [30]	Yoga	(1) Performance quality rating scale (10 pt. scale) (*estimate values*) (2) Canadian occupational performance measure (3) Stroke impact scale (4) Stanford self-efficacy for managing chronic disease (6-item scale) (5) Activity-specific balance confidence	*N* = 3	Rehabilitation center 1 year after stroke Toronto, Canada	Single subject study with 2 replications CO-OP intervention conducted over ~10 sessions	Intervention was associated with significant performance improvements in self-selected functional goals

Johansson et al., 2012 [31]	MBSR Treated/control	(1) Self-assessment of mental fatigue (MBSR) (2) Comprehensive psychopathological rating scale (3) Digit symbol-coding (4) FAS verbal fluency test (5) Trail making test, mental fatigue, and information processing speed	*N* = 29 Stroke (*N* = 18) TBI, (*N* = 11) MBSR (*N* = 12) Wait list control (*N* = 14)	1 year post stroke or TBI patients USA	RCT 8-week MBSR	MBSR may be a promising nonpharmacological treatment for mental fatigue after a stroke or TBI

Hofer et al., 2012 [32]	Yoga	Mental fatigue and related symptoms after neurological disorders and injuries (SQfMF)	*N* = 8	Stroke patients University Hospital of Bern	Single subject study MBCT	Significant pre- to postassessment differences were observed in patients in poststroke fatigue

John et al., 2010 [33]	Group A (film/music) Group B (meditation) Group C (control)	(1) Hamilton rating scale for depression (2) Berg balance scale (3) Barthel ADL index (4) Fatigue severity scale	*N* = 60	Stroke patients Saudi Arabia	RCT 6-week intervention Pre- and postcontrol group design	Music therapy and meditation are more beneficial than conventional physiotherapy management alone

Van Puymbroeck et al., 2012 [34]	Yoga/wait list (control)	Stroke survivor's quality of life (SSQOL)	Yoga (*N* = 37) WL control (*N* = 10)	6 months since last stroke USA	RCT 3 : 1 ratio 8-week yoga intervention	Results showed improved activity, participation, and quality of life relative to controls

nr: not reported, WL: Wait list, RCT: randomized control trial, MBSR: mindfulness-based stress reduction program, MBCT: mindfulness-based cognitive therapy.

## Appendix

For more details, see Figure 1.
